# Effect of Bond Strength of Chitosan Containing Cavity Disinfectant on Deep or Superficial Dentin

**DOI:** 10.3390/biomimetics11080524

**Published:** 2026-07-24

**Authors:** Derya Gursel Surmelioglu, Zeyneb Merve Ozdemir, Songul Ozdogen, Derya Dogan Evlice, Sevim Atilan Yavuz

**Affiliations:** 1 Department of Restorative Dentistry, Faculty of Dentistry, Gaziantep University, Sehitkamil, Gaziantep 27310, Türkiye; 2 Department of Restorative Dentistry, Faculty of Dentistry, Kahramanmaraş Sutcu Imam University, Onikisubat, Kahramanmaraş 46050, Türkiye; zeynebmerveozdemir@ksu.edu.tr; 3 Department of Restorative Dentistry, Faculty of Dentistry, Harran University, Şanlıurfa 63300, Türkiye; ozdogens@gmail.com; 4 Department of Prosthodontics Dentistry, Faculty of Dentistry, Gaziantep University, Sehitkamil, Gaziantep 27310, Türkiye; deryadeniz1538@gmail.com; 5 Department of Restorative Dentistry, Faculty of Dentistry, Mersin University, Mersin 33343, Türkiye; dtsevimatilan@gmail.com

**Keywords:** chitosan, chlorhexidine, microtensile bond strength

## Abstract

Chlorhexidine (CHX) has been widely reported as an inhibitor of matrix metalloproteinases (MMPs), contributing to the preservation of dentin bond integrity, while chitosan (CS) has been proposed as a natural biomodifier with potential collagen-stabilizing and cross-linking effects on the dentin matrix. These properties make both agents relevant candidates for improving the durability of adhesive interfaces. This in vitro study evaluated the effect of CS- and CHX-containing cavity disinfectants on the microtensile bond strength (µTBS) of a two-step self-etch adhesive applied to superficial and deep dentin. Forty-eight sound human molars were randomly assigned to superficial and deep dentin groups, which were further subdivided according to surface treatment: control (no treatment), 2% CHX, and 2.5% CS. A two-step self-etch adhesive system (Clearfil SE Bond) and resin composite were applied according to the manufacturers’ instructions. After storage and thermocycling, beam-shaped specimens were prepared and subjected to µTBS testing. Data were analyzed using appropriate statistical methods, and significance was set at *p* < 0.05. CS pretreatment resulted in higher µTBS values compared with the control group in both superficial and deep dentin, with statistically significant differences observed (*p* < 0.05), while CHX-treated groups showed intermediate values, with no consistent statistically significant difference compared with CS. In general, superficial dentin exhibited higher bond strength values than deep dentin (*p* < 0.05). Within the limitations of this in vitro study, chitosan-containing cavity disinfectant was associated with significantly higher µTBS values compared with the untreated control in both superficial and deep dentin when used with a two-step self-etch adhesive. However, these findings should be interpreted with caution and should not be generalized beyond the specific materials and experimental conditions evaluated.

## 1. Introduction

Hybrid layers formed by adhesives degrade in aqueous environments through hydrolysis of resins and demineralized collagen [1]. Incomplete resin infiltration exposes collagen fibrils at the hybrid layer base, leading to collagen breakdown and resin degradation [2]. The collagen matrix in a dentin layer contains proteases like matrix metalloproteinases (MMPs) and cysteine cathepsins that accelerate collagen degradation [3,4]; however, acidic etchants in bonding procedures expose these endogenous proteases [5,6]. Poor adhesive penetration into collagen fibrils activates enzymes like MMP-2, MMP-9, and MMP-8, affecting hybrid layer formation [3,4]; thus, inhibitory agents are applied to enhance stability through collagen cross-linking [3,7].

Chlorhexidine (CHX) has been widely reported as a nonspecific inhibitor of matrix metalloproteinases (MMPs), contributing to the preservation of dentin bond integrity by reducing enzymatic degradation within the hybrid layer [8,9,10]. It is commonly used at concentrations ranging from 0.12% to 2% and is also incorporated into restorative materials due to its broad-spectrum antimicrobial activity against both Gram-positive and -negative bacteria [11]. However, its concentration-dependent cytotoxicity requires careful optimization to balance its biological safety with its anti-MMP efficacy [12,13]; moreover, CHX cannot co-polymerize with resin monomers, leading to its gradual release from the polymerized resin network, which raises concerns regarding the long-term sustainability of its anti-proteolytic effects [14]. In addition, chitosan has been proposed as a natural biomodifier with promising collagen-stabilizing and cross-linking effects on the dentin matrix. These properties make both CHX and chitosan relevant candidates for enhancing the durability and longevity of adhesive interfaces.

Chitosan (CS) biopolymer effectively reduces collagen matrix degradation by MMPs [7] and, as a β-(1-4) glucosamine derived from chitin in crustacean shells [15], is advantageous due to its non-toxicity, biocompatibility, biodegradability, and antimicrobial properties [16]. Dentin chelation with CS reduces collagen degradation and improves resin–dentin bond durability [17], while CS in restorations enhances antibacterial efficacy and reduces secondary caries risk.

Although the effects of chlorhexidine and chitosan on dentin bonding and matrix metalloproteinase (MMP) inhibition have been widely investigated, most of the available evidence has been obtained using etch-and-rinse (total-etch) adhesive systems [3,5]. In these systems, the aggressive demineralization induced by phosphoric acid exposes collagen fibrils and enhances enzymatic activity, thereby increasing the potential impact of MMP inhibitors [5]. In contrast, self-etch adhesive systems induce a milder and more superficial demineralization pattern, which may result in different interactions with the dentin substrate and reduced MMP activation [3]. Therefore, the effectiveness of cavity disinfectants such as chlorhexidine and chitosan under self-etch conditions remains less clearly defined. In addition, dentin depth is a well-recognized factor influencing bonding performance: superficial dentin typically exhibits lower tubule density and greater intertubular dentin compared with deep dentin, which may favor micromechanical retention and result in higher bond strength values [18]. However, studies directly comparing the effects of cavity disinfectants on both superficial and deep dentin using a standardized self-etch adhesive approach remain limited. Furthermore, although both chlorhexidine and chitosan have been associated with MMP inhibition and collagen stabilization, their combined or comparative effects within the same experimental design have not been sufficiently explored [3,7].

## 2. Materials and Methods

A power analysis was executed employing G*Power (3.1.9.4) software (Heinrich-Heine-Universität Düsseldorf, Düsseldorf, Germany), with parameters set at alpha = 0.05, effect size = 0.45, and power = 0.80, and sample size was determined as 72 specimens.

### 2.1. Sample Preparation

Upon receiving ethical approval, a total of twenty-four human third molar teeth were preserved in a 0.5% chloramine T solution at a temperature of 4 °C for a duration of one month to achieve disinfection. The selection criteria encompassed orthodontic extractions, the absence of caries or restorations, and donors within the age range of 18–25 years to reduce variability in dentin aging. Informed consent was duly obtained from all participants. The roots were excised 1 mm below the cementoenamel junction (CEJ), and the occlusal enamel was meticulously removed horizontally to a level of 1.5 mm and 2.5 mm above the CEJ for deep and superficial dentin, respectively, utilizing a water-cooled diamond disk (Isomet Low-Speed Saw; Buehler Ltd., Lake Bluff, IL, USA). The exposed dentin surface for each tooth was then ground with wet 600-grit SiC paper for 60 s to establish a smear layer, and the pulp was extracted without inflicting damage on the surrounding dentin. The remaining dentin thickness was recorded for deep (0.5–0.9 mm) and superficial dentin (1.5–1.8 mm) [18]. Prior to examination, specimens were stored in distilled water at 37 °C.

### 2.2. Experimental Design

The specimens were allocated into two primary dentin-depth categories—superficial and deep dentin (*n* = 12 per category)—which were then randomly subdivided into three experimental conditions: control, CS, and CHX (*n* = 4). For microtensile testing, three composite–adhesive–dentin slabs were prepared from each specimen; sectioning procedures yielded a total of twelve sticks per subgroup.

To prepare the chitosan (CS) solution, 0.2 g of CS powder (Sigma-Aldrich, St. Louis, MO, USA) was weighed using a precision analytical balance (Sartorius AG, Göttingen, Germany; TE 214 S) and dissolved in 100 mL of 1% acetic acid. The solution was stirred continuously with a magnetic stirrer (RCT Basic, IKA-Werke GmbH & Co. KG, Staufen, Germany) until complete dissolution, resulting in a 0.2% (*w*/*v*) CS formulation used as a cavity disinfectant [19,20].

**Group dC:** Deep dentin surfaces received the self-etch adhesive system strictly according to the manufacturer’s instructions (*n* = 12).

**Group dCS:** Prior to adhesive application, deep dentin was conditioned with 0.2% CS for 60 s and gently blot-dried using absorbent paper. The self-etch adhesive was then applied per the manufacturer’s protocol (*n* = 12).

**Group dCHX:** Deep dentin was pretreated with 2% chlorhexidine (CHX) solution for 60 s, blot-dried, and subsequently bonded using the self-etch adhesive as recommended by the manufacturer (*n* = 12).

**Group sC:** Superficial dentin surfaces were bonded with the self-etch adhesive following the manufacturer’s instructions (*n* = 12).

**Group sCS:** Superficial dentin was treated with 0.2% CS for 60 s, was blot-dried, and then received the self-etch adhesive according to the manufacturer’s guidelines (*n* = 12).

**Group sCHX:** Superficial dentin was pretreated with 2% CHX for 60 s, blot-dried, and bonded using the same self-etch adhesive protocol (*n* = 12).

The two-step self-etch adhesive, Clearfil SE Bond (Kuraray, Tokyo, Japan), was applied to the dentin surface, air-dried for 2 s, and subsequently cured with a Valo LED (Ultradent Products Inc., South Jordan, UT, USA) for a duration of 10 s. The Clearfil Majesty Posterior resin composite was then incrementally layered, each approximately 2 mm in thickness, until reaching a total thickness of 4 mm; each layer underwent polymerization for 20 s. Following this procedure, the teeth were immersed in artificial saliva at 37 °C for a period of 24 h before being subjected to thermal cycling.

### 2.3. Thermal Cycles

Samples were stored at 37 °C in distilled water for 24 h, then thermocycled (Thermocycler, SD Mechatronik, Feldkirchen Westerham, Germany) for 10,000 cycles between 5 and 55 °C with 30 s dwelling and 10 s transit times [21].

### 2.4. Microtensile Bonding Strength (μTBS) Test

Deep and superficial dentin specimens were sectioned perpendicular to the dentin–resin interface under continuous water irrigation using a low-speed precision saw (Isomet 1000, Buehler Ltd., Lake Bluff, IL, USA). This procedure produced beam-shaped sticks with an approximate cross-sectional dimension of 1 × 1 mm, the thickness and dimensions of which were measured and confirmed with a digital caliper prior to testing (Mitutoyo, Tokyo, Japan).

Specimens were secured to the testing jig (Odeme Dental Research, Luzerna, SC, Brazil) with a cyanoacrylate adhesive (Patex; Henkel) and subjected to µTBS testing using a dedicated device (Micro Tensile Tester, BISCO Inc., Schaumburg, IL, USA). Test load was applied under displacement control at a crosshead speed of 0.5 mm/min and continued until failure occurred. Bond strength values were reported in megapascals (MPa) and computed as the ratio of the maximum failure load (kgf) to the bonded cross-sectional area (cm^2^) of each beam, which had been measured prior to testing.

### 2.5. Failure Mode Analysis

Failure types were identified using a stereomicroscope (Leica Microsystems, Wetzlar, Germany) with a magnification setting of ×25. The classification was based on the predominant failure mode, categorized as follows: (a) adhesive failure, which occurs at the interface between the resin-based restoration and the dentin surface; (b) cohesive failure, which takes place within the resin-based restoration itself; (c) mixed failure, which involves a combination of adhesive and cohesive fractures.

### 2.6. Statistical Analysis

Data analysis was performed utilizing SPSS version 22 for Windows (IBM Corp., Armonk, NY, USA). The assumptions of normality and homogeneity of variances were verified through the Kolmogorov–Smirnov and Levene’s tests, respectively, while the chi-squared test was employed to examine the types of failure. The μTBS test groups were compared using ANOVA, followed by Tukey’s post hoc tests for multiple comparisons.

## 3. Results

### 3.1. μTBS Results

Table 1 displays the mean and standard deviations (SDs) of the μTBS values.

For superficial dentin, the highest mean bond strength was observed in the sCS group (44.6 ± 3.3), followed by the sCHX group (33.6 ± 3.1), while the lowest values were recorded in the control group.

For deep dentin, the highest mean bond strength was observed in the dCS group (40.6 ± 3.4), whereas the lowest values were recorded in the dC group (29.4 ± 3.2).

In general, bond strength values were higher in superficial compared to deep dentin across all treatment groups.

Statistical analysis revealed significant differences among groups depending on both dentin depth and surface treatment (*p* = 0.032). Post hoc comparisons indicated that CS-treated groups showed significantly higher bond strength values compared with the control group in both superficial and deep dentin, while CHX-treated groups showed intermediate values.

### 3.2. Mode of Failure Results

Table 2 illustrates the types of failure observed in the μTBS. The most prevalent failure type was adhesive failure, occurring in 23 out of 72 specimens, accounting for 31.9% of the total; cohesive failures were observed in 25 specimens (34.7%), while mixed fractures were noted in 24 (33.3%) across all groups.

## 4. Discussion

Cavity disinfectants are commonly used in dental practice to eliminate microorganisms prior to restorative procedures [22,23]. The present in vitro study evaluated the effect of CHX- and CS-containing cavity disinfectants on µTBS in superficial and deep dentin.

The results showed CS had higher bonding strength than CXH and control groups in both superficial and deep dentin, while CHX and CS showed similar results—both performing better than control groups. Both null hypotheses were accepted. The μTBS was higher in superficial than deep dentin across all disinfectants. CS application as cavity disinfectant improved composite–dentin bonding through collagen preservation and enhanced interfacial compatibility [24].

The outcomes of the present study are in agreement with some previous studies that assessed the effect of different cavity disinfectants on the bond strength of resin-based composite restorations to dentin. For instance, Surmelioglu et al. [25] found that CS cavity disinfectant increased the μTBS of composite restorations to both irradiated and non-irradiated dentin compared to CHX and control groups. Similarly, Mohamed et al. [26] reported that chitosan nanoparticles improved the bond strength of resin composite to dentin. Coelho et al. [27] conducted a systematic review and concluded that CHX cavity disinfectant could preserve adhesion to dentin compared to the control group, while Ziotti et al. [28] showed that CS-treated specimens had higher µTBS than the control group to both sound and demineralized dentin. Paschoini et al. [29] demonstrated that a CS-treated dentin surface using either an etch-and-rinse or self-etch adhesive system improved the immediate bond strength of the adhesive interface and maintained it for up to 6 months. Another study demonstrated that CHX exhibited superior bonding to self-etch adhesive compared to the control group [29].

However, contradictory findings have also been reported. AlSheikh et al. [30] found that CS exhibited lower bond strength under certain conditions, potentially due to interference with polymerization or hybrid layer formation. Similarly, CS nanoparticles showed lower bond strength than CHX in some studies [31]. Dacoreggio et al. [32] reported that CS may reduce µTBS depending on adhesive strategy, possibly due to tubule obliteration that limits resin infiltration. These discrepancies may be related to differences in concentration, application protocols, dentin characteristics, and adhesive systems.

On the other hand, a significant decrease was detected in the etch-and-rinse method, interpreted by the obliteration of the dentinal tubules, which prevents adequate resin infiltration, despite the electrostatic interaction of CS with the organic component of demineralized dentin. This suggested that CS might harmfully influence the polymerization of adhesive resin or interfere with the hybrid layer formation. The disagreement among study results may depend on factors like disinfectant type and concentration, application method, dentin characteristics, adhesive system protocol, and bond strength testing; therefore, standardized methods are needed to assess cavity disinfectants’ influence on composite–dentin bond strength.

Regarding CHX concentration, conflicting findings have also been reported: lower concentrations (e.g., 1%) have been associated with improved bond strength, whereas 2% CHX may reduce it under certain conditions [33]; in contrast, other studies have shown no adverse effect of 2% CHX when used with etch-and-rinse systems [10].

In our study, it was found that superficial dentin has higher bond strength than deep dentin, regardless of the cavity disinfectant used [34]. The reason for this is that the former has more hydroxyapatite crystals and less collagen fibers than the latter, which affects the bonding mechanism and durability [35]. Furthermore, superficial dentin has less bacterial contamination and MMP activity than deep dentin, which reduces the risk of secondary caries and bond breakdown [35,36].

The bond strength between the tooth and the restorative material depends not only on the numerical data, but also on the fracture pattern on the adhesive surface. If the fracture happens in the weakest part of the tooth or the material, it means that the stress was evenly distributed, and the true adhesive bond values cannot be measured. Therefore, it is important to identify which adhesive fracture type can best reflect the bond strength [37]. In our research, we discovered that all the groups had more adhesive than cohesive fractures. Failure mode analysis revealed a distribution of adhesive, cohesive, and mixed failures without a clearly dominant pattern across all groups, suggesting that bond strength values cannot be attributed to a single failure mechanism.

It should be noted that most studies investigating the effects of chlorhexidine and chitosan on dentin bonding have been conducted using etch-and-rinse adhesive systems [3,5]. In such systems, the aggressive demineralization caused by phosphoric acid exposes collagen fibrils and increases the activation of endogenous MMPs, thereby enhancing the potential role of MMP inhibitors [5]. In contrast, self-etch adhesive systems induce a more superficial demineralization and partially preserve the mineral phase surrounding collagen fibrils, which may result in reduced MMP activation [3]; consequently, the effectiveness of MMP inhibitors such as chlorhexidine and chitosan may differ under self-etch conditions. Therefore, evaluating these agents within a self-etch adhesive strategy, as performed in the present study, provides relevant information that cannot be directly extrapolated from studies based on etch-and-rinse systems. Furthermore, the improved bond strength observed after CS or CHX pretreatment may be associated with mechanisms previously reported in the literature, such as protease inhibition or collagen-related interfacial stabilization [3,5], while that observed in CS and CHX groups may also be associated with mechanisms such as enhanced surface wettability, modification of the smear layer, and interactions with collagen fibrils, as suggested in previous studies [3,7]. It has been suggested that CHX and chitosan do not necessarily form a physical barrier that prevents resin infiltration; instead, they may interact with the dentin surface without significantly impairing monomer penetration, allowing for the formation of resin tags within the dentin substrate [3,5]. However, the present study did not directly assess these mechanisms; therefore, such explanations should be interpreted with caution [5].

The present study has several inherent limitations.

First, a two-step self-etch adhesive system was employed instead of an etch-and-rinse approach, which is often associated with higher immediate bond strength. Additionally, bond performance was evaluated solely using the µTBS test; therefore, no complementary analyses were performed to investigate underlying mechanisms such as MMP activity, hybrid layer morphology, or nanoleakage. The inclusion of additional techniques, such as scanning electron microscopy or nanoleakage assessment, could have provided further insight into interfacial characteristics.

Second, only sound dentin was used as the bonding substrate. Although this approach improves standardization and reproducibility in adhesion studies [38], it does not fully represent clinical conditions. Caries-affected dentin, which is commonly encountered in clinical practice, exhibits structural and compositional differences that may influence bonding performance [39,40]. Accordingly, using sound dentin allows for a more controlled evaluation of the direct influence of cavity disinfectants on adhesive performance, without the additional uncertainties introduced by caries-related alterations. Nevertheless, it should be emphasized that the bonding behavior of sound dentin is not identical to that of caries-affected dentin: the latter commonly yields reduced bond strength and less favorable hybrid layer characteristics, potentially due to differences in mineral-to-matrix ratio, crystallinity, and collagen organization [39,40]. Therefore, while the use of sound dentin provides methodological standardization, it also limits the direct clinical extrapolation of the findings.

Third, multiple microtensile sticks obtained from the same tooth were treated as independent observations. Although this approach is commonly used in µTBS studies, it may introduce pseudoreplication and overestimate statistical power [41,42,43].

Furthermore, the relatively small number of teeth per subgroup represents an additional limitation. Although multiple sticks were obtained from each tooth, these do not constitute independent biological samples, and thus the effective sample size remains limited [42,43]. A power analysis was performed to assess the adequacy of the sample size; however, the limited number of teeth may still restrict the biological representativeness and generalizability of the results.

Finally, the findings of the present study are restricted to the specific experimental conditions employed, including the use of a single self-etch adhesive system, evaluation of sound dentin, and a short-term in vitro aging protocol. Bonding performance is influenced by adhesive strategy, dentin substrate characteristics, and aging conditions [1,2,3]; therefore, the results cannot be directly extrapolated to etch-and-rinse systems, universal adhesives applied under different strategies, caries-affected dentin, or long-term clinical performance. In addition, the in vitro design cannot fully replicate clinical conditions; hence, further in vivo studies are required to evaluate the long-term performance of cavity disinfectants. Accordingly, all findings should be interpreted with caution.

## 5. Conclusions

Within the limitations of this in vitro study, pretreatment with a chitosan-containing cavity disinfectant was associated with higher µTBS values than the untreated control in both superficial and deep dentin when used with the tested self-etch adhesive. However, in vitro bond strength outcomes do not necessarily reflect long-term clinical performance, as bonding effectiveness is influenced by multiple biological and environmental factors. Therefore, the present findings should be interpreted with caution and should not be generalized beyond the specific materials and experimental conditions evaluated.

## Figures and Tables

**Table 1:** Bond strength of study groups (Mean ± SD).

	Groups	MPa (Mean ± SD)
**Deep Dentin**	Group dC (Clearfil SE Bond)	29.4 ± 3.2 ^a^
	Group dCS (CS + Clearfil SE Bond)	40.6 ± 3.4 ^b^
	Group dCHX (CHX + Clearfil SE Bond)	39.1 ± 3.6 ^b^
**Superficial Dentin**	Group sC (Clearfil SE Bond)	33.6 ± 3.1 ^c^
	Group sCS (CS + Clearfil SE Bond)	44.6 ± 3.3 ^d^
	Group sCHX (CHX + Clearfil SE Bond)	42.0 ± 6.1 ^bd^

Different letters within the lines indicate statistically significant differences. CHX: chlorhexidine diguanite. CS: chitosan. Group dC: Control group in deep dentin surface + Clearfil SE Bond; Group dCS: CS-treated deep dentin + Clearfil SE Bond; Group dCHX: CHX-treated deep dentin + Clearfil SE Bond; Group sC: Control group in surface dentin + Clearfil SE Bond; Group sCS: CS-treated surface dentin + Clearfil SE Bond; Group sCHX: CHX-treated surface dentin + Clearfil SE Bond.

**Table 2:** Types and frequency of bonding failure for each group (%).

	Group dC	Group dCS	Group dCHX	Group sC	Group sCS	Group sCHX	Total Frequency Percent
**Adhesive**		4/12 (33%)	3/12 (25%)	4/12 (33.3%)	4/12 (33%)	5/12 (41.6%)	23/72 (31.9%)
**Cohesive** **Dentin**	6/12 (50%)	5/12 (41.6%)	5/12 (41.6%)	4/12 (33.3%)	2/12 (16.6%)	3/12 (25%)	25/72(34.7%)
**Mixed**	3/12 (25%)	3/12 (25%)	4/12 (33.3%)	4/12 (33.3%)	6/12 (50%)	4/12 (33.3%)	24/72(33.3%)

CHX: chlorhexidine diguanite. CS: chitosan. Group dC: Control group in deep dentin surface + Clearfil SE Bond; Group dCS: CS-treated deep dentin + Clearfil SE Bond; Group dCHX: CHX-treated deep dentin + Clearfil SE Bond; Group sC: Control group in surface dentin + Clearfil SE Bond; Group sCS: CS-treated surface dentin + Clearfil SE Bond; Group sCHX: CHX-treated surface dentin + Clearfil SE Bond.

## Data Availability

The data that support the findings of this study are available on request from the corresponding author.

## Abbreviations

The following abbreviations are used in this manuscript:

CEJ	cementoenamel junction
CHX	chlorhexidine
CS	chitosan
MMPs	matrix metalloproteinases
